# Coverage and equity in reproductive and maternal health interventions in Brazil: impressive progress following the implementation of the Unified Health System

**DOI:** 10.1186/s12939-016-0445-2

**Published:** 2016-11-17

**Authors:** Giovanny V. A. França, María Clara Restrepo-Méndez, Maria Fátima S. Maia, Cesar G. Victora, Aluísio J. D. Barros

**Affiliations:** International Center for Equity in Health, Federal University of Pelotas, Rua Marechal Deodoro, 1160 3° Piso, 96020-220 Pelotas, RS Brazil

**Keywords:** Maternal health, Health inequalities, Socioeconomic factors, Brazil

## Abstract

**Background:**

The Brazilian SUS (Unified Health System) was created in 1988 within the new constitution, based on the premises of being universal, comprehensive, and equitable. The SUS offers free health care, independent of contribution or affiliation. Since then, great efforts and increasing investments have been made for the system to achieve its goals. We assessed how coverage and equity in selected reproductive and maternal interventions progressed in Brazil from 1986 to 2013.

**Methods:**

We reanalysed data from four national health surveys carried out in Brazil in 1986, 1996, 2006 and 2013. We estimated coverage for six interventions [use of modern contraceptives; antenatal care (ANC) 1+ visits by any provider; ANC 4+ visits by any provider; first ANC visit during the first trimester of pregnancy; institutional delivery; and Caesarean sections] using standard international definitions, and stratified results by wealth quintile, urban or rural residence and country regions. We also calculated two inequality indicators: the slope index of inequality (SII) and the concentration index (CIX).

**Results:**

All indicators showed steady increases in coverage over time. ANC 1+ and 4+ and institutional delivery reached coverage above 90 % in 2013. Prevalence of use of modern contraceptives was 83 % in 2013, indicating nearly universal satisfaction of need for contraception. On a less positive note, the proportion of C-sections has also grown continuously, reaching 55 % in 2013. There were marked reductions in wealth inequalities for all preventive interventions. Inequalities were significantly reduced for all indicators except for the C-section rate (*p* = 0.06), particularly in absolute terms (SII).

**Conclusions:**

Despite the difficulties faced in the implementation of SUS, coverage of essential interventions increased and equity has improved dramatically, due in most cases to marked increase in coverage among the poorest 40 %. An increase in unnecessary Caesarean sections was also observed during the period. Further evaluation on the quality of healthcare provided is needed.

## Background

Social inequalities are still a major problem in Latin America in spite of recent economic growth [1, 2]. Health inequalities are also a leading health problem in the region [1–3]. In response to these challenges, substantial increases in public funding for social programmes and the adoption of relevant policies and strategic health-related initiatives took place in the region over the past few decades [3, 4]. Brazil provides an example of a distinct approach to health-system reform, combining poverty reduction strategies with the expansion of comprehensive primary health care services [5, 6]. Hence, lessons from the Brazilian experience are relevant for countries advancing in universal health coverage.

The health sector reform in Brazil is unique because it was driven by civil society rather than by governments, political parties, or international organizations [5, 7]. It was designed by militants of the so-called Sanitary Movement nearly a decade before health care was incorporated in the 1998 Constitution as a citizen’s right and State’s duty [7–9]. In the 1990’s health sector reform was institutionalised with the creation of a national Unified Health System (SUS, *Sistema Único de Saúde*), based on the principles of universality, equity, integrality and social participation [5, 8].

Since then, the SUS became a driving force for equalizing access to services by offering free and comprehensive health care for all, independent of contribution or affiliation [5, 9]. Other advances during the past 20 years included investments in human resources, primary care, science and technology, decentralization, widespread social participation, and growing public awareness of a right to health care. Among the many programs and policies put in place since the inception of SUS, two are of major importance. First, the Family Health Program changed the structure of traditional primary health care centres, adding community health workers and defining catchment areas for facilities. The program was targeted at the poorest, under-served urban neighbourhoods and rural areas [10]. Later, the *Programa Bolsa Família*, a conditional cash transfer program, unified several social benefit initiatives in one large program designed to provide extra cash to the poorest families in the country, conditional on use of health services by children and on regular school attendance. The program was successful in reducing income inequality [11], contributing to a significant decrease in childhood mortality, especially from poverty-related causes such as malnutrition and diarrhea [12]. In parallel, the country went through a period of rapid economic changes, with the control of hyperinflation in 1994, and strong economic growth between 2004 and 2011.

Despite criticisms regarding underfunding and poor management of the SUS, as well as of the unequal distribution of power and resources, [8] there is mounting evidence that health inequalities in Brazil have been steadily declining [5]. Since 1990’s, Brazil has increased intervention coverage in maternal and child interventions, reduced inequalities in terms of under-5 mortality and practically eliminated inequalities in stunting [2]. However, it is worth noting that most of this evidence is based on the Brazilian household survey conducted in 2006, and therefore, no reliable population-based data on maternal and child health has been available for the last 9 years. In this article, we describe how coverage and inequalities in reproductive and maternal health interventions evolved over time, based on the analyses of four national household surveys carried out from 1986 to 2013, covering a period of nearly three decades.

## Methods

### Study design and data sources

The International Centre for Equity in Health (ICEH, www.equidade.org) monitors equity in health and nutrition by reanalysing population-based surveys, especially the Demographic and Health Surveys (DHS) and Multiple Indicator Cluster Surveys (MICS). Our analyses relied on data from the following national surveys carried out in Brazil: Demographic Health Surveys – 1986 [13] and 1996 [14]; Pesquisa Nacional de Demografia e Saúde (PNDS), a survey similar to DHS carried out in 2006, funded by the Ministry of Health [15]; and the National Health Survey (PNS) carried out in 2013 by the Brazilian Statistics Office (IBGE) and the Oswaldo Cruz Foundation [16].

### Selection of indicators

Out of a set of 80 reproductive, maternal, newborn and child health (RMNCH) indicators routinely calculated by the ICEH, data were available for six reproductive and maternal interventions for at least three out of the four surveys under study. We applied the following definitions:

- Use of modern contraceptives: percentage of women age 15–49 years currently married or in union who are using (or whose partner is using) a modern contraceptive method;
- Antenatal care 1+ visits (ANC 1+): percentage of women aged 15–49 with a live birth in the survey reference period who had one or more ANC-related visits during pregnancy, by any provider;
- Antenatal care 4+ visits (ANC 4+): percentage of women aged 15–49 with a live birth in the survey reference period who had four or more ANC-related visits during pregnancy, by any provider;
- First ANC visit during the first trimester of pregnancy: percentage of women aged 15–49 with a live birth in the survey reference period who underwent the first prenatal visit during the first trimester of pregnancy;
- Institutional delivery: percentage of live births which took place in a health facility;
- C-sections rate: percentage of live births delivered by Caesarean section.

Survey reference periods included births in the past 5 years in the first three surveys, and in the past 2 years for the 2013 survey.

### Data analyses

All analyses were carried out in Stata (StataCorp. 2013. Stata Statistical Software: Release 13. College Station, TX: StataCorp LP), taking into account the survey design, including sampling weights and clustering. All point estimates of coverage and inequality indices were calculated with standard errors, based on the original data sets. Indicators were disaggregated by household wealth quintiles based on asset indices, urban and rural residence and country region. Asset indices [17] were calculated by the DHS team for the 1996 survey; for the other three surveys, we used similar methods to derive asset indices using principal component analyses based on household goods, characteristics of the house and available infrastructure, such as types of water access and sanitation facilities. Asset indices were grouped into quintiles, with Q1 representing the poorest and Q5 the wealthiest 20 % of households.

### Measures of inequalities

We calculated two inequality indicators that take the whole distribution of wealth into account: the slope index of inequality (SII) and the concentration index (CIX). The SII expresses the absolute difference in percentage points between the projected coverage for the top and the bottom of the wealth distribution, [18] using a logistic regression model. The CIX is based on concept similar to the Gini index for income concentration, being expressed on a scale from −100 to +100, with zero representing equal distribution of the attribute across the wealth scale. Positive CIX values represent a pro-rich distribution, usually observed for health coverage indicators. The SII expresses absolute inequality, whereas the CIX expresses relative inequality [18, 19].

### Time trends

Variance-weighted least squares regression was used to estimate the average absolute annual change in coverage and in inequality measures taking into account the time intervals between surveys, allowing tests of the statistical significance of the observed trends. Survey year was used as the independent variable in the time trend analyses. Annual changes were estimated at the national level, for the poorest (Q1) and richest (Q5) quintiles, and for CIX and SII. Absolute changes are expressed in percentage points per year. Results were omitted when the unweighted number of observations in a specific subgroup was less than 25.

### Ethics

We used publicly available data from national surveys in our analyses. Ethical clearance was obtained by the institutions that carried out the surveys. Further information about the surveys and ethics can be found in their respective websites [20–23]. The PNS project was approved by the National Commission of Ethics in Research (CONEP) in June 2013, Regulation No. 328.159.

## Results

**Fig. 1:**
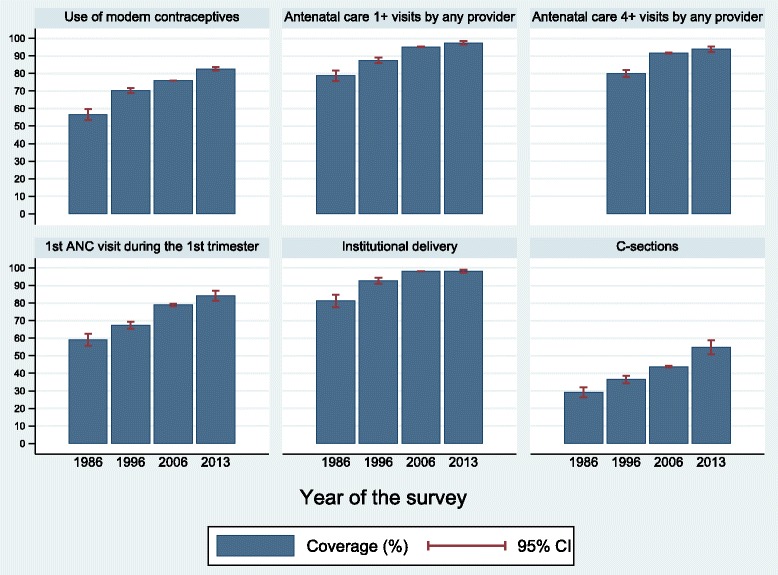
National coverage of six reproductive and maternal interventions, Brazil, from 1986 to 2013. Source: Brazil DHS 1986, DHS 1996, PNDS 2006, PNS 2013

We were able to calculate the selected indicators for all surveys, except for ANC 4+ visits in the 1986 survey in which the number of visits was not recorded. Figure 1 shows the national coverage of the six interventions (Table 1). All indicators showed steady increase in coverage over the years. ANC 1+ and 4+ and institutional delivery reached coverage above 90 % in 2013. Prevalence of use of modern contraceptives was 83 % in 2013, what corresponds to 95 % of family planning coverage (percent of women in need that are using contraception) according to a recently published paper (REF). On a less positive note, the proportion of C-sections has also grown continuously, reaching 55 % in 2013.

**Table 1:** Coverage of six reproductive and maternal interventions by geographic region, Brazil, from 1986 to 2013

Indicator	Year	National			Area of residence					
					Urban			Rural		
		%	95 % CI		%	95 % CI		%	95 % CI	
Use of modern contraceptives	1986	56.6	53.6	59.6	63.6	60.9	66.3	43.3	37.1	49.4
	1996	70.3	68.9	71.7	72.6	71.2	74.1	61.2	57.8	64.6
	2006	75.9	75.8	76.0	76.0	75.9	76.2	75.5	75.1	75.9
	2013	82.6	81.6	83.6	83.0	81.9	84.0	80.3	77.6	82.9
Antenatal care 1+ visits (any provider)	1986	78.7	75.7	81.8	88.2	85.5	91.0	56.8	50.9	62.7
	1996	87.5	85.9	89.1	92.0	90.7	93.2	72.0	66.9	77.0
	2006	95.2	95.0	95.4	96.4	96.3	96.6	89.5	86.4	92.6
	2013	97.4	96.5	98.4	97.4	96.3	98.5	97.5	95.7	99.3
Antenatal care 4+ visits (any provider)	1986	NA	NA	NA	NA	NA	NA	NA	NA	NA
	1996	79.9	77.9	82.0	86.0	84.2	87.8	59.1	53.2	65.1
	2006	91.7	91.4	92.0	93.5	93.3	93.7	83.4	80.1	86.8
	2013	93.9	92.3	95.5	94.5	92.9	96.1	90.6	85.3	95.9
1st ANC visit during the 1st trimester	1986	59.1	55.8	62.5	69.2	65.7	72.8	35.9	31.0	40.7
	1996	67.4	65.4	69.5	72.7	70.8	74.7	49.3	44.4	54.1
	2006	78.9	78.2	79.6	79.6	78.7	80.4	75.8	74.1	77.5
	2013	84.3	81.4	87.1	85.3	82.3	88.4	78.6	70.6	86.6
Institutional delivery	1986	81.2	77.7	84.8	92.0	89.8	94.2	59.8	53.4	66.1
	1996	92.7	91.1	94.3	96.9	96.0	97.8	79.6	74.4	84.7
	2006	98.1	98.1	98.2	98.7	98.7	98.7	95.8	95.6	96.1
	2013	98.1	97.3	99.0	99.2	98.7	99.8	92.4	88.1	96.7
C-sections	1986	29.2	26.3	32.0	36.2	33.1	39.4	15.1	11.4	18.8
	1996	36.4	34.2	38.7	41.8	39.2	44.3	20.1	16.3	23.9
	2006	43.8	43.3	44.3	45.9	45.3	46.5	35.2	32.6	37.9
	2013	54.7	50.7	58.7	58.2	53.8	62.6	36.0	28.0	44.0

Source: Brazil DHS 1986, DHS 1996, PNDS 2006, PNS 2013. *NA* not available, *95 % CI* 95 % confidence interval

**Fig. 2:**
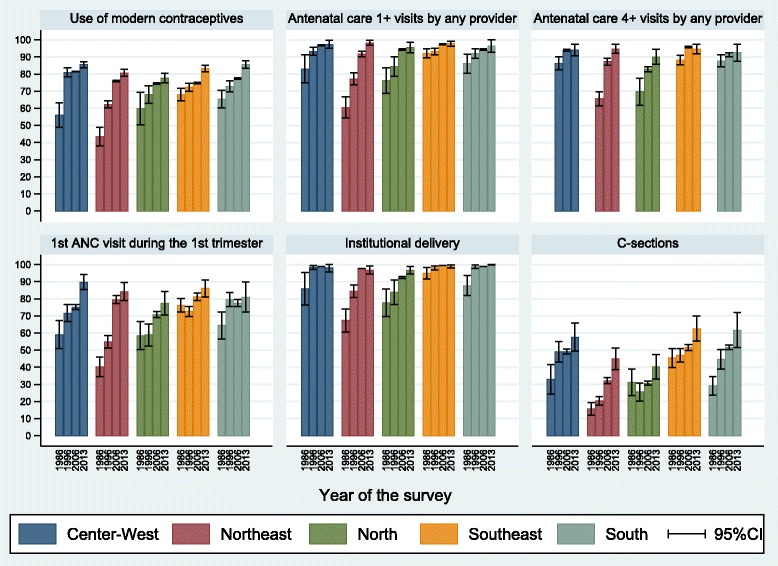
Coverage of six reproductive and maternal interventions by geographic region, Brazil, from 1986 to 2013. Source: Brazil DHS 1986, DHS 1996, PNDS 2006, PNS 2013

Coverage trends for geographic regions are presented in Fig. 2 (Table 2). The Northeast and North regions showed marked increases for most indicators over time. By 2013 these regions reached coverage levels similar to those in the wealthiest regions (Southeast and South), except for the C-section rate. The North region is slightly behind in terms of ANC visits in the first trimester.

**Table 2:** Coverage of six reproductive and maternal interventions by geographic region, Brazil, from 1986 to 2013

Indicator	Year	Region of the country														
		Center-West			North			Northeast			Southeast			South		
		%	95 % CI		%	95 % CI		%	95 % CI		%	95 % CI		%	95 % CI	
Use of modern contraceptives	1986	55.9	48.8	63.1	59.8	50.3	69.2	43.3	37.9	48.7	68.1	64.3	71.8	65.5	60.4	70.6
	1996	81.0	78.3	83.7	68.1	62.9	73.3	62.3	60.2	64.4	72.4	70.0	74.7	72.7	69.5	75.9
	2006	81.5	81.3	81.7	74.4	73.9	74.9	75.9	75.5	76.2	74.7	74.3	75.1	77.3	77.0	77.7
	2013	85.5	83.8	87.3	77.7	75.0	80.4	80.7	78.7	82.7	83.2	81.5	85.0	85.6	83.4	87.7
Antenatal care 1+ visits (any provider)	1986	83.1	74.9	91.2	76.3	68.9	83.7	60.5	54.4	66.6	92.1	89.4	94.8	86.1	80.5	91.7
	1996	93.2	90.9	95.5	84.3	78.6	90.0	77.2	73.6	80.7	93.1	91.2	95.0	92.0	89.2	94.8
	2006	96.8	96.4	97.2	94.3	93.8	94.8	91.8	90.2	93.4	97.3	97.0	97.7	94.4	94.0	94.8
	2013	97.3	95.0	99.7	95.4	92.3	98.5	98.2	96.8	99.7	97.8	96.4	99.2	96.3	92.7	99.9
Antenatal care 4+ visits (any provider)	1986	NA	NA	NA	NA	NA	NA	NA	NA	NA	NA	NA	NA	NA	NA	NA
	1996	86.3	82.3	90.2	69.7	61.8	77.6	65.5	61.5	69.6	88.2	85.4	91.0	87.8	84.1	91.4
	2006	93.8	93.2	94.4	82.8	81.4	84.2	87.2	85.2	89.1	95.8	95.4	96.3	91.3	90.0	92.5
	2013	94.0	90.8	97.3	90.0	85.7	94.4	94.8	92.0	97.5	94.7	92.0	97.4	92.5	87.4	97.5
1st ANC visit during the 1st trimester	1986	59.1	51.0	67.2	58.5	50.4	66.7	40.3	34.5	46.1	76.3	72.2	80.3	64.5	56.6	72.4
	1996	71.6	66.6	76.6	58.9	52.5	65.4	54.9	51.2	58.6	72.7	69.7	75.6	79.6	75.4	83.8
	2006	75.2	73.6	76.7	70.8	69.0	72.5	79.5	77.2	81.8	81.1	78.9	83.3	77.5	75.5	79.5
	2013	89.8	85.3	94.2	77.5	70.6	84.4	84.3	79.0	89.5	86.1	81.2	91.0	80.9	72.2	89.7
Institutional delivery	1986	86.0	76.5	95.4	77.7	69.6	85.9	67.4	60.6	74.1	95.0	91.5	98.4	87.7	81.9	93.6
	1996	98.3	97.2	99.4	83.8	76.7	90.9	84.3	80.7	88.0	98.1	96.8	99.3	98.8	97.7	99.9
	2006	98.8	98.8	98.8	92.5	91.9	93.0	97.7	97.6	97.8	99.5	99.5	99.5	99.0	98.9	99.0
	2013	98.1	95.7	100.0	96.7	94.5	98.8	96.8	94.5	99.2	98.9	97.9	99.8	99.9	99.7	100.0
C-sections	1986	33.0	24.4	41.5	31.3	23.6	39.0	15.7	12.1	19.3	45.4	39.8	51.0	29.2	23.8	34.7
	1996	49.1	43.1	55.0	25.5	20.3	30.8	20.4	18.0	22.7	46.9	42.9	50.9	44.6	38.9	50.2
	2006	49.2	47.9	50.5	30.7	29.6	31.8	32.2	30.4	34.0	51.7	49.9	53.4	51.7	50.4	53.0
	2013	57.6	49.5	65.7	40.2	33.1	47.4	45.0	38.6	51.3	62.6	55.2	70.0	61.7	51.4	71.9

Source: Brazil DHS 1986, DHS 1996, PNDS 2006, PNS 2013. *NA* not available, *95 % CI* 95 % confidence interval

**Fig. 3:**
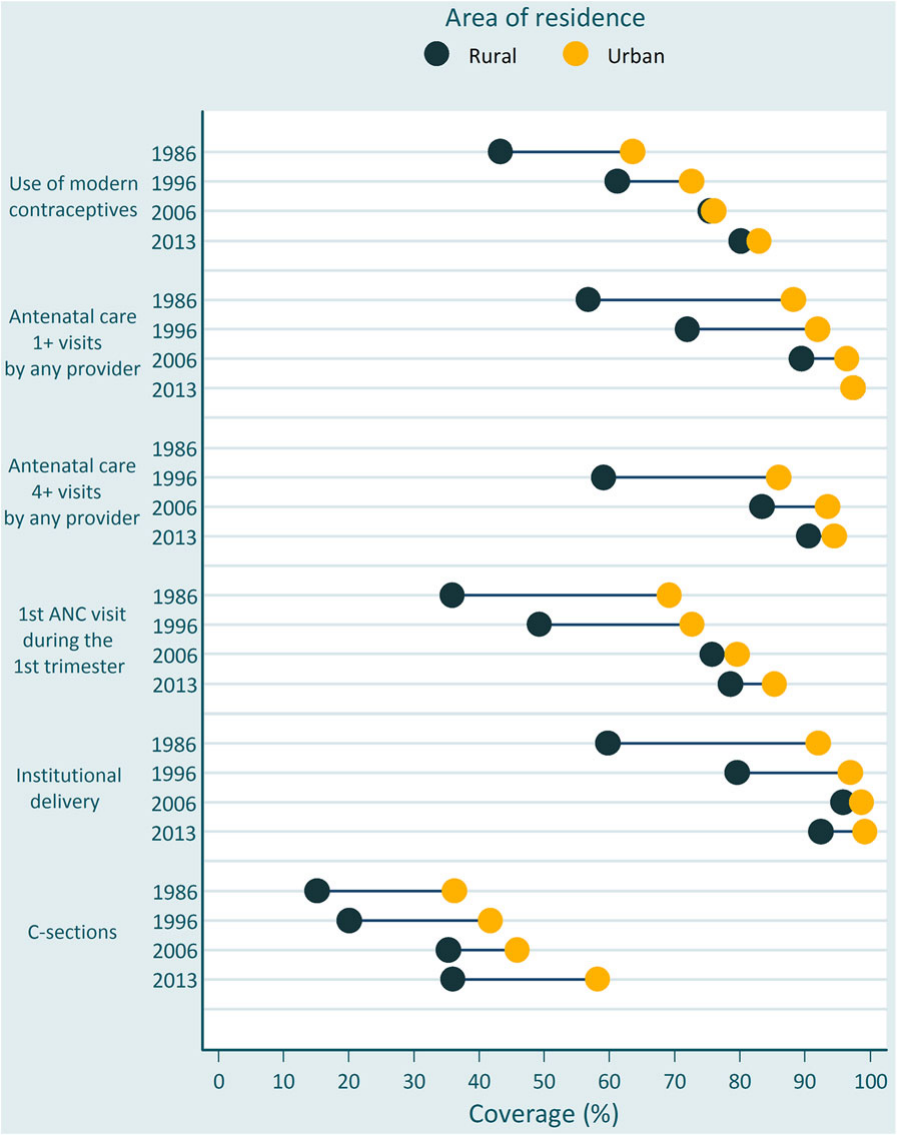
Coverage of six reproductive and maternal interventions by area of residence, Brazil, from 1986 to 2013. Source: Brazil DHS 1986, DHS 1996, PNDS 2006, PNS 2013. *Coloured dots* show the average coverage in each category (urban/rural). *Horizontal lines* connect the average coverage in urban (*yellow circles*) and rural (*dark green circles*) areas. The distance between the dots represents absolute inequality. The longer the line between the two groups, the greater the absolute inequality

Figure 3 (Table 1) shows coverage trends in urban and rural areas. Urban coverage was substantially larger in 1986. These differences were attenuated and virtually disappeared by 2013 for use of modern contraceptives, ANC 1+ and ANC 4+ visits. Regarding C-sections, the increase between 2006 and 2013 was restricted to urban women, thus widening the urban-rural gap (58.3 % vs. 36.0 %).

**Fig. 4:**
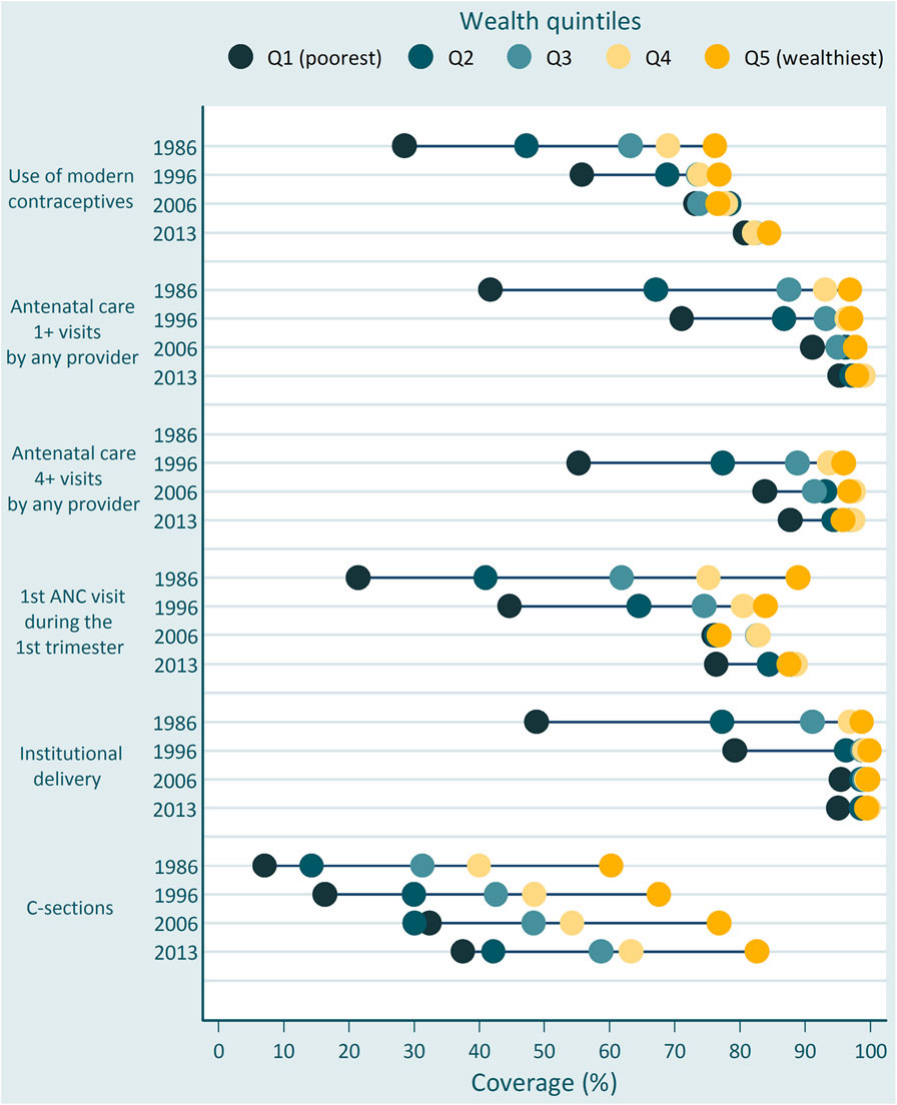
Coverage of six reproductive and maternal interventions by wealth quintiles, Brazil, from 1986 to 2013. Source: Brazil DHS 1986, DHS 1996, PNDS 2006, PNS 2013. *Coloured dots* show the average coverage in each wealth quintile. Q1 is the 20 % poorest wealth quintile; Q5 is the 20 % richest. The distance between quintiles 1 and 5 represents absolute inequality

Figure 4 present coverage by wealth over time. Each circle represents coverage in each wealth quintile. Horizontal lines connect the poorest and wealthiest quintiles, with longer lines representing larger absolute inequalities. There were large reductions in inequalities for all interventions. Use of contraceptives, ANC and institutional deliveries presented bottom inequality early on [19] with the poorest 20 % being well behind the other groups. Disparities in C-section rates were those with the smallest reduction (Table 3).

**Table 3:** Coverage of six reproductive and maternal interventions by wealth quintiles and magnitude of inequalities, Brazil, from 1986 to 2013

Indicator	Year	Wealth quintiles															Inequality measures					
		Q1 (poorest)			Q2			Q3			Q4			Q5 (20 % richest)			CIX			SII		
		%	IC95 %		%	IC95 %		%	IC95 %		%	IC95 %		%	IC95 %		%	IC95 %		%	IC95 %	
Use of modern contraceptives	1986	28.5	23.3	33.8	47.3	41.6	53.0	63.2	59.1	67.4	69.0	64.7	73.3	76.2	72.9	79.5	17.1	54.0	59.1	55.6	50.1	63.1
	1996	55.8	52.8	58.7	68.9	66.2	71.7	73.6	71.0	76.3	73.8	71.2	76.5	76.8	74.1	79.5	5.3	69.2	71.3	22.3	65.9	74.7
	2006	73.2	72.9	73.6	78.4	78.0	78.8	73.9	73.3	74.4	77.8	77.2	78.4	76.6	75.9	77.3	1.0	74.8	77.1	3.2	70.5	81.4
	2013	80.9	78.8	83.0	82.3	80.1	84.5	82.3	79.9	84.8	82.3	79.9	84.6	84.6	82.6	86.5	0.8	81.9	83.3	3.9	79.3	85.9
Antenatal care 1+ visits (any provider)	1986	41.7	35.9	47.5	67.2	62.3	72.1	87.6	84.4	90.8	93.1	90.3	96.0	97.0	95.5	98.5	13.5	76.9	80.5	64.4	72.5	84.9
	1996	71.1	67.2	75.1	86.9	84.2	89.5	93.2	91.1	95.3	96.5	94.5	98.5	97.0	95.5	98.6	6.7	86.4	88.6	36.6	81.4	93.5
	2006	91.1	89.6	92.7	96.2	96.0	96.5	95.1	93.6	96.5	97.9	97.7	98.1	97.7	97.5	98.0	1.5	94.0	96.4	8.2	87.4	100.0
	2013	95.4	92.9	97.8	97.2	94.5	99.9	98.6	97.5	99.7	99.0	97.7	100.0	98.0	96.5	99.6	0.7	96.8	98.0	4.5	93.2	100.0
Antenatal care 4+ visits (any provider)	1986	NA	NA	NA	NA	NA	NA	NA	NA	NA	NA	NA	NA	NA	NA	NA	NA	NA	NA	NA	NA	NA
	1996	55.3	50.9	59.7	77.4	74.0	80.9	88.8	86.3	91.4	93.7	91.3	96.2	96.0	94.3	97.7	11.4	78.5	81.4	54.0	74.2	85.7
	2006	83.8	81.8	85.9	93.1	92.5	93.8	91.5	89.8	93.2	97.5	97.3	97.7	96.9	96.5	97.3	3.1	90.1	93.2	17.0	82.5	100.0
	2013	87.7	83.2	92.3	94.5	91.2	97.7	97.1	95.5	98.8	97.4	94.9	99.8	95.9	92.7	99.0	2.0	92.6	95.1	12.4	86.1	100.0
1^st^ ANC visit during the 1^st^ trimester	1986	21.4	16.2	26.6	41.0	35.4	46.7	61.9	57.9	65.9	75.2	70.2	80.2	88.9	85.8	92.1	23.2	57.0	61.2	74.1	54.3	63.9
	1996	44.7	41.0	48.4	64.5	60.9	68.2	74.6	70.8	78.3	80.6	77.0	84.1	83.9	80.2	87.7	12.6	65.9	69.0	48.9	61.9	73.0
	2006	76.0	74.4	77.7	76.5	73.6	79.4	82.7	80.6	84.9	82.8	80.5	85.1	76.9	72.6	81.1	1.7	76.6	81.2	6.3	67.9	90.0
	2013	76.4	70.0	82.9	84.6	79.5	89.7	87.9	82.5	93.4	88.6	83.1	94.1	87.6	80.4	94.8	3.1	81.9	86.6	15.4	72.9	95.6
Institutional delivery	1986	48.8	41.1	56.4	77.4	73.1	81.6	91.2	88.1	94.2	97.0	95.2	98.8	98.8	97.7	99.8	12.6	79.0	83.5	64.2	72.6	89.9
	1996	79.3	75.2	83.4	96.3	94.9	97.7	98.9	98.2	99.6	99.2	98.4	99.9	100.0			5.3	91.5	93.9	41.7	83.7	100.0
	2006	95.6	95.4	95.7	98.7	98.6	98.7	99.1	99.1	99.2	99.5	99.4	99.5	99.7	99.7	99.7	1.0	97.7	98.6	5.6	95.3	100.0
	2013	95.1	92.4	97.8	98.6	97.0	100.0	99.4	98.7	100.0	99.7	99.2	100.0	99.4	98.6	100.0	1.1	97.5	98.8	7.8	92.0	100.0
C-section	1986	7.1	4.3	10.0	14.3	10.9	17.6	31.3	26.9	35.8	40.0	34.1	45.9	60.4	55.9	64.8	36.3	25.7	32.6	61.2	23.5	34.8
	1996	16.3	13.9	18.8	30.0	26.6	33.4	42.6	38.5	46.7	48.5	44.1	53.0	67.7	63.0	72.4	27.9	34.1	38.8	57.2	31.7	41.2
	2006	32.5	30.5	34.4	30.1	28.8	31.3	48.4	46.4	50.5	54.3	51.5	57.1	76.9	74.3	79.4	18.8	39.8	47.9	45.2	34.4	53.2
	2013	37.5	31.3	43.7	42.2	34.6	49.9	58.7	50.2	67.3	63.3	52.7	73.9	82.6	76.3	89.0	17.2	51.1	58.3	53.5	44.6	64.8

Source: Brazil DHS 1986, DHS 1996, PNDS 2006, PNS 2013. *NA* not available, *95 % CI* 95 % confidence interval

**Fig. 5:**
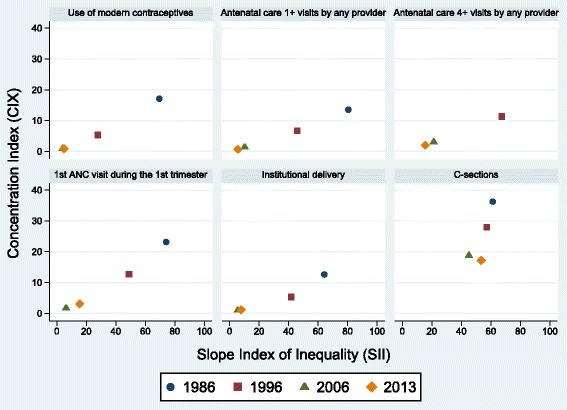
Scatter diagrams of concentration index and slope index of inequality for six reproductive and maternal interventions, Brazil, from 1986 to 2013. Source: Brazil DHS 1986, DHS 1996, PNDS 2006, PNS 2013

Figure 5 shows scatter diagrams of changes in CIX and SII. According to absolute inequalities (SII), the first ANC visit during the first trimester of pregnancy was the most inequitable intervention in 1986, but substantial progress was achieved over time. In 2013, the most inequitable intervention was C-section, which showed little progress in terms of absolute inequalities. Substantial decreases in relative inequalities (CIX) were achieved for all indicators. Although the CIX for C-section was reduced by half from 1986 to 2013, it remained as the most inequitable intervention in relative terms (Table 3).

Significant increases in overall coverage were found for all indicators, as well as for the poorest and wealthiest 20 % for most indicators (Table 4). In contrast, the antenatal and delivery care indicators did not increase over time among the 20 % wealthiest women. Absolute and relative equity improved significantly for nearly all indicators with greater reductions in terms of absolute than of relative inequality, except for C-sections rate (*p* = 0.06).

**Table 4:** Annual changes in coverage and inequality measures in six reproductive and maternal interventions, Brazil, from 1986 to 2013

Indicator	Group		Average annual change		
			Coefficient	SE	*p*-value
Use of modern contraceptives	Overall		0.82	0.04	<0.001
	Coverage	Q1 (poorest)	1.74	0.08	<0.001
		Q5 (wealthiest)	0.25	0.06	<0.001
	Inequality measures	CIX	−0.37	0.03	<0.001
		SII	−1.63	0.12	<0.001
Antenatal care 1+ visits (any provider)	Overall		0.61	0.04	<0.001
	Coverage	Q1 (poorest)	1.77	0.10	<0.001
		Q5 (wealthiest)	0.04	0.03	0.42
	Inequality measures	CIX	−0.41	0.03	<0.001
		SII	−2.19	0.13	<0.001
Antenatal care 4+ visits (any provider)	Overall		0.79	0.08	<0.001
	Coverage	Q1 (poorest)	2.06	0.19	<0.001
		Q5 (wealthiest)	0.06	0.08	0.48
	Inequality measures	CIX	−0.55	0.06	<0.001
		SII	−2.59	0.28	<0.001
1st ANC visit during the 1st trimester	Overall		1.02	0.06	<0.001
	Coverage	Q1 (poorest)	2.63	0.11	<0.001
		Q5 (wealthiest)	−0.36	0.11	<0.001
	Inequality measures	CIX	−0.81	0.05	<0.001
		SII	−2.57	0.19	<0.001
Institutional delivery	Overall		0.34	0.04	<0.001
	Coverage	Q1 (poorest)	1.29	0.11	<0.001
		Q5 (wealthiest)	0.03	0.02	0.18
	Inequality measures	CIX	−0.31	0.03	<0.001
		SII	−2.38	0.18	<0.001
C-sections	Overall		0.78	0.06	<0.001
	Coverage	Q1 (poorest)	1.28	0.08	<0.001
		Q5 (wealthiest)	0.84	0.11	<0.001
	Inequality measures	CIX	−0.73	0.09	<0.001
		SII	−0.46	0.19	0.06

Source: Brazil DHS 1986, DHS 1996, PNDS 2006, PNS 2013

*SE* standard error, *SII* slope index of inequality, *CIX* concentration index

## Discussion

In spite of its economic growth up to the recent past, Brazil is still among the five Latin American countries with the greatest income inequalities [24, 25]. Nevertheless, our findings show improved equity in access to health services as can be seen by increased coverage in reproductive and maternal health interventions and the remarkable reductions in geographic and wealth-related inequalities. Even the poorest 20 % of women and those living in rural or remote areas achieved near universal coverage levels with preventive interventions by 2013. To date, this is the first study to examine the evolution of coverage and inequalities of a relevant set of maternal health interventions covering a period of three decades and including the most recent data from national household surveys in Brazil. In addition, this article presents the most complete evidence of universal coverage achievement in terms of maternal health interventions.

In terms of improving equity, the best possible combination is when both absolute and relative indices improve; this was the case Brazil for all selected interventions, that shows important progress across all quintiles of wealth index and also faster progress among the poorest 20 %.

Several factors seem to have contributed to increasing coverage and reducing inequalities, [6], including economic growth, reduction in income inequality, urbanisation, improved education, and decreased fertility. The control of hyperinflation in 1994, modernization of the Brazilian economy, high Gross Domestic Product (GDP) growth rates between 2004 and 2011, and increased social investment contributed to this positive scenario. Anti-poverty actions such as the conditional cash transfer programme (Programa Bolsa Família) are likely to have contributed to the changes.

Within the health sector, the creation of the tax-funded national health system (SUS) in 1988 extended free health care to a significant proportion of the population that was excluded until then, mostly rural workers and the unemployed or informal workers [5, 9]. Before the inception of SUS, Brazil’s healthcare system was based on private organizations that received large government subsidies. An important program was the Family Health Program (*Programa Saúde da Família – PSF)*, established in the mid-1990s, which expanded the primary health care network to reach the poorest areas of the country. Its innovative approach of starting with areas that were devoid of any services, and the inclusion of community health workers to the health team was enormously successful, with its coverage expanding rapidly since its inception, reaching 55 % in 2012 [3, 4, 6, 9]. The SUS also includes a National Immunisation Programme (PNI) and the *Farmácia Popular*, a program that delivers free or heavily subsided medicines for diabetes, hypertension, asthma, and other common diseases through accredited private pharmacies [5, 26].

The Brazilian healthcare system is a mix of public and private services, and users are free to choose between them. Public health services are provided mostly by public facilities at the primary care level, and by private and philanthropic hospitals at tertiary level. The system is financed through direct taxes and social contributions [5, 26]. Public funding for the SUS has been steadily increasing over the years in both absolute values and in proportion of GDP. The percentage of the GDP spent on health increased from 7.2 % in 2000 to 9.5 % in 2012, in addition, the government funding accounted for 47.5 % of the expenditure on health in 2012.

The public health expenditure share of the GDP in all levels of government – federal, state and municipal - increased from 2.9 to 3.9 % between 2000 and 2011 [27]. Despite these advances, the public health expenditure in Brazil is still much lower compared to other countries with universal health systems [28]. Total health expenditures per capita have also increased steadily over the years; however, the government still accounted for less than 50 % of total health expenditure by 2012 [29]. The remainder results from a combination of out-of-pocket and private insurance spending, which is among the highest levels of private spending on health in Latin America [30].

Additional evidence shows that out-of-pocket spending as a proportion of total spending varies little between the poorest and wealthiest classes [5, 31]. For instance, the catastrophic health expenditure (10 % or more of capacity to pay based on household consumption) was 18.4 % from the poorest and 17.7 % for the wealthiest in 2008–2009 [32]. However, rich and poor spend these funds in different ways. Among the latter, out-of-pocket expenditures are mostly due to purchasing medicines, whereas the richest spend most on private health insurance [5, 31].

Our results on C-section rates confirm the disturbing trends documented by the nationwide information system (DATASUS) [33]. Among the wealthiest quintile the proportion of C-sections was above 80 % in 2013. These rates are unacceptable high considering those recommended by WHO despite several efforts to encourage vaginal deliveries and limit C-sections: payment of delivery analgesia for SUS patients (1998), the Pact for C-section Rate Reduction between the Brazilian Ministry of Health and state health departments (2000), enforcement of a ceiling of 27 % C-section rate for states that did not sign the Pact (2002), and a national mass campaign, “Humanization of Normal Childbirth and Reduction of Unnecessary Cesareans” (2006) [34]. In Brazil, obstetricians assist almost all deliveries regardless of financing or budget constraints, and their convenience may play an important role in the decision about the type of delivery. There is widespread evidence that doctors’ attitudes during the prenatal and peri-delivery period may increase the likelihood of a C-section [34]. Unless strong and immediate action is taken, Brazil is at risk of reaching universal coverage for an intervention which is estimated to be necessary at most for 15 % of all deliveries [35].

A main limitation of our analyses is that some of the surveys failed to employ internationally-standardized questionnaires, so it was not possible to estimate key RMNCH indicators, nor to assess time trends for more than a few indicators. Standardized surveys, carried out every 3–5 years, are essential for monitoring progress and identifying trends in inequalities. It is worth noting that other relevant dimensions of social inequalities in intervention coverage were not assessed, including those associated with women’s schooling or ethnic group.

## Conclusions

In summary, there was enormous progress from 1986 to 2013 for key interventions in reproductive and maternal health, both in terms of coverage and inequality. Disparities in C-section rates remain, but these are due to exceedingly high rates of unnecessary procedures among the rich, rather than low rates among the poor. Dissemination of the lessons learned is Brazil is particularly relevant in light of the focus on universal health coverage and equity which are key aspects of the Sustainable Development Goals (SDGs) of the 2030 Agenda for Sustainable Development adopted by world leaders in September 2015.

Despite such progress, the mortality rates of children and mothers remain several fold higher in Brazil than in the best-performing countries [36, 37] indicating that there is still much room for improvement. Substantial challenges remain for the Brazilian health system, including reforming its financial structure to ensure universality and long term sustainability, renegotiating public and private roles, and assuring quality and efficiency of care while minimizing medicalization and ensuring patient safety – as exemplified by the high C-section rates [5]. In terms of equity, the overarching challenge is how to reach those who are hardest to reach, such as rural populations in the Amazon rainforest and northeast regions, indigenous groups, and families living in Brazilian municipalities where with insufficient human resources for health [6]. Also, ensuring universal access is insufficient unless high-quality care is provided and missed opportunities for promoting other interventions is assured [5, 38, 39]. We hope that our findings will inform policy debates on strategies to achieve universal coverage and to reduce health inequalities in other low- and middle-income countries, and showcase the importance of tracking progress by regularly collecting data.
